# Impacto do serviço de telediagnóstico nas internações por doenças cardiovasculares: uma abordagem em municípios da Bahia, Brasil

**DOI:** 10.1590/0102-311XPT088123

**Published:** 2024-11-04

**Authors:** Thiago Gonçalves do Nascimento Piropo, Francisco de Sousa Ramos

**Affiliations:** 1 Universidade Federal de Pernambuco, Recife, Brasil.

**Keywords:** Doenças Cardiovasculares, Telecardiologia, Eletrocardiografia, Telemedicina, Atenção Primária à Saúde, Cardiovascular Diseases, Telecardiology, Electrocardiography, Telemedicine, Primary Health Care, Enfermedades Cardiovasculares, Telecardiología, Electrocardiografía, Telemedicina, Atención Primaria de Salud

## Abstract

Os gastos com internações por doenças do aparelho circulatório são elevados: em 2019, apenas para o Estado da Bahia, Brasil, excederam 153 milhões de reais para o Sistema Único de Saúde, superando os custos de internações por câncer. Esse fato tende a se acentuar com o aumento da expectativa de vida no Brasil (7,3 anos a mais até 2060). A introdução de novas tecnologias pode mitigar o problema. Este estudo analisa o impacto da utilização do telediagnóstico em eletrocardiograma nas internações por doenças cardiovasculares, em 326 municípios baianos durante o período de 2014 a 2020. O método de estimador *diff-in-diff* foi utilizado para a análise entre os períodos anterior e posterior à implantação do serviço no estado. Os resultados mostram que os municípios que introduziram a nova tecnologia reduziram em seis internações por doenças cardiovasculares a cada ano adicional. No caso das famílias beneficiadas pelo Bolsa Família, a redução foi de 3,26 internações, e de 3,08 entre os municípios com o serviço especializado. Verificou-se um aumento de 7,66 internações na faixa etária de 30 a 59 anos e um aumento de 5,34 entre homens, a cada ano adicional. Os resultados evidenciam uma redução de 1,15 internações por doenças cardíacas reumatológicas e de 1,39 entre as pessoas diabéticas. No quesito raça/cor, foi identificada subnotificação nas condições estudadas, resultando em prognósticos mais severos para a população preta. A tecnologia mostrou-se eficaz para reduzir essa desigualdade, ao expandir o acesso e reduzir as internações, tendo um papel crucial na saúde coletiva e impactando na redução da mortalidade. O tema, portanto, é merecedor de mais estudos, com amostras e períodos amostrais diferentes.

## Abstract (en)

Hospitalization costs due to cardiovascular diseases are high. In 2019, for Bahia State, Brazil, alone, they exceeded BRL 153 million for the Brazilian Unified National Health System, surpassing the costs of cancer hospitalizations. This fact will show an upward trend with the increase in life expectancy in Brazil (7.3 years more by 2060). Introducing new technologies can mitigate the problem. This study analyzes the impact of telediagnostics in electrocardiogram on hospitalizations for cardiovascular diseases in 326 municipalities in Bahia from 2014 to 2020. Diff-in-diff estimator method was used for analysis of the periods before and after the implementation of telediagnostics in Bahia. Results show that the municipalities which introduced the new technology reduced cardiovascular diseases hospitalizations by 6 for each additional year. In the case of families benefiting from the Brazilian Income Transfer Program, the reduction was 3.26 hospitalizations, and 3.08 among municipalities with the specialized service. Hospitalization increase by 7.66 in the 30 to 59 age group and by 5.34 among men for each additional year. Results show a reduction of 1.15 hospitalizations for rheumatologic heart diseases and 1.39 among diabetic people. In terms of ethnicity/color, underreporting was identified in the conditions studied, resulting in more severe prognoses for blacks. Telediagnostics was effective in reducing this inequality by expanding access and reducing hospitalizations, playing a crucial role in public health and impacting mortality reduction. The theme, therefore, deserves further studies with different samples and sample periods.

## Resumen

Los costos de las hospitalizaciones por enfermedades del sistema circulatorio son elevados: en el 2019, solo para el Estado de Bahía, Brasil, superaron los BRL 153 millones para el Sistema Único de Salud, superando los costos de las hospitalizaciones por cáncer. Este hecho tiende a acentuarse con el aumento de la esperanza de vida en Brasil (7,3 años más hasta el 2060). La introducción de nuevas tecnologías puede mitigar este problema. Este estudio analiza el impacto del uso del telediagnóstico por electrocardiograma en las hospitalizaciones por enfermedades cardiovasculares en 326 municipios de Bahía durante el período de 2014-2020. Para el análisis entre los períodos anterior y posterior a la implementación del servicio en el Estado, se utilizó el método de estimador *diff-in-diff*. Los resultados muestran que los municipios que introdujeron la nueva tecnología redujeron las hospitalizaciones por enfermedades cardiovasculares en 6 cada año adicional. En el caso de las familias beneficiarias del Bolsa Familia, la reducción fue de 3,26 hospitalizaciones y de 3,08 entre los municipios con servicio especializado. Se constató un aumento de 7,66 hospitalizaciones en el rango de edad de 30 a 59 años y un aumento de 5,34 entre hombres, por cada año adicional. Los resultados muestran una reducción de 1,15 hospitalizaciones por cardiopatías reumatológicas y de 1,39 entre personas diabéticas. En términos de raza/color, se identificó una infranotificación en las condiciones estudiadas, lo que resultó en pronósticos más severos para la población negra. La tecnología ha demostrado ser eficaz para reducir esta desigualdad, al ampliar el acceso y reducir las hospitalizaciones, desempeñando un papel crucial en la salud colectiva e impactando en la reducción de la mortalidad. El tema, por lo tanto, merece más estudios, con diferentes muestras y períodos muestrales.

## Introdução

Os gastos com internações por doenças do aparelho circulatório são relativamente elevados. Dados do Sistema de Informações Hospitalares (SIH) ^1^ mostram que, em 2019, apenas no Estado da Bahia, Brasil, esses custos excederam 153 milhões de reais para o Sistema Único de Saúde (SUS), superando aqueles com internações por câncer (aproximadamente 120 milhões de reais no mesmo período).

A incidência de doenças cardiovasculares tende a se acentuar em decorrência do aumento da expectativa de vida dos brasileiros: projeções demográficas e epidemiológicas apontam que ainda haverá aumento de 7,3 anos até 2060; evidenciando, em paralelo, uma maior prevalência da carga de doenças crônicas não transmissíveis (DCNT) ^2^, refletindo significativamente na elevação dos gastos públicos em saúde ^3^. Entre essas, as doenças cardiovasculares irão demandar uma melhor organização da Rede de Atenção à Saúde (RAS), incluindo medidas preventivas, diagnóstico precoce, acesso aos tratamentos farmacológicos essenciais e reabilitação ^2^.

Segundo a Organização Pan-Americana da Saúde (OPAS), mais pessoas morrem por complicações com patologias do coração por ano do que qualquer outra enfermidade. Em 2016, as duas causas de óbitos mais frequentes foram a doença cardíaca isquêmica, seguida pelo acidente vascular cerebral (AVC) ^4^. Esse cenário representou 31% das mortes ocorridas em todo o mundo, principalmente em países de baixa e média renda ^5^.

É consenso na literatura que a maioria das doenças cardiovasculares pode ser prevenida por meio do monitoramento e do controle dos fatores de risco já conhecidos, sendo fundamental o diagnóstico e o tratamento precoces ^5^ ^,^ ^6^. A realização de exames permite identificar riscos, investigar enfermidades e orientar para os tratamentos adequados, dado que as doenças cardiovasculares podem ser silenciosas ou confundidas com outras doenças ^6^. O eletrocardiograma (ECG) tem um papel fundamental na identificação das alterações de ritmo cardíaco, bloqueios e sinais de infarto do miocárdio, reduzindo desfechos cardiovasculares e mortalidade precoce e tardia, constituindo-se em um relevante marcador de doenças do coração ^7^. O exame de ECG é uma das medidas preventivas mais utilizadas em cardiologia, devido ao seu baixo custo, à sua fácil execução e por ser uma modalidade não invasiva, com elevada sensibilidade para o diagnóstico de diversas doenças e quadros sistêmicos, e não apenas cardiológicos ^8^.

Apesar da importância do exame de ECG para a intervenção precoce, há uma disparidade quanto ao seu acesso nos serviços públicos e privados de saúde em todo o país, sobretudo para as pessoas de baixa renda, de baixa escolaridade, pretas e situadas nas regiões Norte e Nordeste, conforme dados da *Pesquisa Nacional de Saúde* (PNS) de 2019 ^9^. Esses dados não surpreendem: iniquidades em saúde constituem um dos traços mais acentuados da situação nacional ^7^, com elevados índices de vulnerabilidade social, morbidades e óbitos evitáveis, resultado da exclusão ou da limitação do acesso de pessoas ou grupos aos serviços e equipamentos públicos ^10^.

A PNS de 2019 ^9^ revelou que o percentual de pessoas que nunca mediram a pressão arterial é maior entre os homens e duas vezes maior entre pessoas autodeclaradas pretas, comparativamente às autodeclaradas brancas. Além disso, o percentual das que tiveram o diagnóstico médico para alguma doença cardiovascular foi menor entre pessoas pretas, bem como a solicitação do exame de ECG durante as consultas com médico especialista, mesmo tendo apresentado algum sinal clínico ^9^. Ainda nessa linha, constata-se que o ECG foi menos solicitado entre as pessoas com menor grau de instrução, internadas por hipertensão arterial sistêmica ou complicações. Ademais, as pessoas autodeclaradas pretas sofrem mais por limitações intensas provocadas por alguma doença cardíaca. Finalmente, o maior percentual de pessoas que nunca mediram o colesterol está nas regiões Norte e Nordeste do Brasil (23% e 18,1%, respectivamente) ^9^.

Esses contrastes constituem um perfil epidemiológico preocupante, envolvendo dimensões socioeconômicas, educacionais, geográficas, de saneamento básico, ocupação, gênero, raça/cor e oferta de equipamentos nos diferentes grupos populacionais. Como consequência, pessoas com doenças cardiovasculares e outras doenças não transmissíveis são diagnosticadas mais tardiamente e apresentam o risco de morte prematura aumentada para idade produtiva ^4^ ^,^ ^11^ ^,^ ^12^.

Uma das estratégias que tem avançado nos últimos anos, e com potencial para a melhoria do cuidado, é a incorporação de tecnologias digitais voltadas para saúde ^13^. Com o advento de novas tecnologias, como a possibilidade de transmissão de dados de saúde pela internet, amplia-se a capacidade de difusão, principalmente em regiões carentes do país, fornecendo um melhor padrão de atendimento assistencial, absolutamente necessário e fundamental no diagnóstico de doenças cardiovasculares e em diversas situações clínicas ^14^. Uma das áreas de destaque é a telecardiologia, ou telemedicina cardiológica, particularmente o telemonitoramento da atividade cardíaca por meio do ECG ^15^.

Pesquisas já apontam para uma redução dessa mortalidade, principalmente nas pequenas cidades onde inexistem unidades de tratamento especializado, com o uso de inovações em telecardiologia e com a realização do exame de ECG ao nível local, transmitido via internet para ser interpretado por um especialista, que disponibiliza o laudo do ECG e orienta a equipe de saúde quanto ao manejo adequado do paciente em poucos minutos ^16^.

Estudos sobre os impactos dessa intervenção na qualidade de vida das pessoas com alguma doenças cardiovasculares são escassos ^16^. Revisões sistemáticas recentes, com metanálise, investigaram a efetividade da transmissão de dados em ECG, com foco em pacientes com insuficiência cardíaca, sendo possível destacar a redução no número de internações e mortalidade, quando comparados a métodos convencionais do cuidado ^17^ ^,^ ^18^ ^,^ ^19^ ^,^ ^20^.

A fim de verificar qual o impacto da utilização do telediagnóstico em ECG nas internações por doenças cardiovasculares, este estudo aborda o caso dos municípios baianos que implantaram o serviço de telediagnóstico em ECG. O serviço é gerenciado pelo Ministério da Saúde e executado, na Bahia, pelo Núcleo Técnico-Científico de Telessaúde, tendo o Centro de Telemedicina de Minas Gerais, Hospital das Clínicas, Universidade Federal de Minas Gerais, como responsável por realizar os laudos à distância, prestando apoio especialmente aos municípios de pequeno porte, com menos recursos tecnológicos e clínica especializada ^21^. A técnica utilizada é o método conhecido como *diff-in-diff* (diferenças-em-diferenças).

## Métodos

Trata-se de um estudo quase-experimental, não randomizado, baseado em pré e pós-intervenção, descritivo e analítico, com dados secundários e abordagem quantitativa. Para analisar os dois períodos, o anterior à implantação do serviço de telediagnóstico em ECG no Estado da Bahia e o posterior a essa implantação, utilizou-se o estimador *diff-in-diff*. Considera-se dois momentos: (1) 2014 a 2017, pré-implantação do serviço; e (2) 2018 a 2020, pós-implantação. Esses períodos representam 108 mil exames de ECG laudados.

Portanto, dos 417 municípios baianos, tem-se 326 municípios na amostra: 23 deles compõem o grupo de tratamento, com laudos solicitados em 2018 e monitorados até 2020; e os 303 restantes compõem o grupo de controle (municípios que não implantaram o serviço). O total de laudos solicitados por meio da Plataforma Nacional de Telediagnóstico (PNTD) é de 59.852 laudos de ECG reconhecidos para o período.

O serviço foi implantado na Bahia em novembro de 2017. Entretanto, para fins deste estudo, será tomado como critério de inclusão os municípios com implantação do serviço até o ano de 2018, devido ao maior quantitativo. Aqueles que descontinuaram o serviço durante esse período foram excluídos. Ressalta-se que foi considerado com serviço implantado o município treinado e cadastrado na PNTD, a partir da realização do exame de ECG ao nível local e da solicitação do primeiro laudo realizado por especialistas, enviado via internet.

Para a classificação dos municípios adotou-se a definição do Instituto Brasileiro de Geografia e Estatística (IBGE), segundo tipologia rural-urbana, que considera, além da densidade populacional, o acesso dos municípios a bens e serviços mais complexos, e sua localização ou a acessibilidade aos centros urbanos mais estruturados ^22^. Sendo assim, 74% dos municípios com o serviço implantado são do tipo predominantemente rural adjacente.

Os dados utilizados provêm das bases secundárias do SIH e do Sistema de Informações Ambulatoriais (SIA) do Ministério da Saúde. Os dados geográficos e populacionais foram obtidos do IBGE, e o número de solicitação de laudos por meio da PNTD. Informações complementares foram obtidas a partir de legislação específica e de diretrizes publicadas por organizações (inter)nacionais.

A variável independente é a internação por doenças cardiovasculares; as demais variáveis de agravos se referem ao número de internações no SUS por 100 mil habitantes, extraídos do SIH, e informações de saúde epidemiológicas e morbidade, por local de residência.

As variáveis de controle de serviços caracterizaram a oferta de programas e serviços de saúde oferecidos à população, por município, como: taxa de cobertura populacional estimada das Equipes de Saúde da Família; número de Núcleos de Apoio a Saúde da Família e Academia da Saúde; número total de pessoas acompanhadas pelo Sistema de Vigilância Alimentar e Nutricional (SISVAN); número de ECG convencionais realizados; e a quantidade de serviços de atenção cardiovascular com atendimento pelo SUS por município.

As variáveis de controle de agravos apresentam o respectivo número de internações, segundo as principais condições de saúde em cardiologia, no período - a exemplo do infarto agudo do miocárdio, da insuficiência cardíaca, da arritmia cardíaca, do acidente vascular cerebral, da hipertensão arterial sistêmica e da diabetes mellitus -, segundo o município, por local de residência, entre outros. Finalmente, as variáveis de controle socioeconômico evidenciam o perfil da população (gênero, faixa etária, raça/cor, características durante o internamento hospitalar, valores, taxa de mortalidade e número de óbito) acometida por alguma doença cardiovascular.

Todos os municípios foram identificados pelos respectivos códigos na *Pesquisa de Informações Básicas Municipais* (MUNIC) do IBGE e, para a análise estatística e econométrica, utilizou-se o software R (http://www.r-project.org), com o RStudio, versão 4.1.2 (https://rstudio.com/) - *Bird Hippie*.

O método de estimador *diff-in-diff* foi utilizado para a avaliação da análise entre os dois períodos, anterior e posterior à implantação do serviço no estado, compondo o consolidado relativo de implantações do serviço de telecardiologia em ECG iniciadas em 2018. A abordagem de *diff-in-diff* para dois grupos e dois períodos oferece algumas vantagens: (1) proporciona uma análise econométrica transparente e gera estimativas empíricas que são rapidamente interpretáveis; (2) a validade das hipóteses de identificação pode ser mais diretamente avaliada; e (3) exige pouca estrutura dos parâmetros sobre o problema ^22^.

A utilização do método *diff-in-diff* baseia-se na hipótese de que a trajetória temporal da variável em foco para o grupo de controle representa o que teria ocorrido na ausência de tratamento ^23^. O efeito da intervenção é então capturado pela diferença da diferença dos resultados, para os grupos de tratamento e controle, antes e depois da política/programa. No caso, os municípios não adotantes do serviço (grupo de controle) e os adotantes (grupo de tratamento) apresentariam a mesma tendência nas taxas de internações que as do grupo de tratamento na ausência de implantação do serviço, considerados os fatores observáveis.

Após a intervenção, espera-se um desvio na linha de tendência do grupo de tratamento, retratando o impacto provocado pela adoção do serviço (período pós-intervenção), eliminando dessa maneira, os efeitos de fatores não observados que possam afetar o resultado, mas que sejam constantes ao longo do tempo e entre os grupos ^24^.

Ressalte-se que este trabalho utilizou apenas dados secundários, não oferecendo risco de qualquer natureza, conforme *Resolução nº 510/2016* do Conselho Nacional de Saúde. Portanto, não necessitou ser encaminhado ao Comitê de Ética em Pesquisa.

## Resultados

A análise dos dados (Tabela 1) mostra o baixíssimo número médio dos serviços especializados no estado (0,67), bem como a média de estabelecimentos que realizam o exame de ECG tradicional (2,17). Outro ponto refere-se às solicitações de ECG: a média de realização do exame com laudo enviado pela internet (69,41) já supera a média dos exames com laudso locais (49,3), mesmo considerando o formato *online* aplicado para apenas 23 municípios.

**Tabela 1:** Descritivo das variáveis de controle de serviços oferecidos para a população dos municípios do Estado da Bahia, Brasil (2014 a 2020).

Variáveis	Observações	Média	DP	Mínimo	Máximo
Serviços de atenção cardiovascular com atendimento pelo SUS *	2.919	0,67	4,42	0	105
Estabelecimentos que realizam exames de ECG convencionais **	2.919	2,17	11,72	0	244
Laudos realizados em telecardiologia *	1.668	69,41	311,13	0	7.167
Exames de ECG convencionais realizados **	2.919	49,30	251,53	0	5.233
Taxa de cobertura populacional das equipes de saúde da família *	2.905	92,77	14,07	0	100
Núcleos de Apoio a Saúde da Família (tipos 1,2 ou 3) implantados *	2.919	0,24	0,50	0	5
Academias da Saúde *	2.919	0,34	0,56	0	3
Pessoas acompanhadas pelo SISVAN (gestantes, adultos e idosos)**	2.919	418,57	329,95	4	1.679,9
Pessoas com sobrepeso/algum grau de obesidade, acompanhadas pelo SISVAN (gestantes, adultos e idosos) **	2.919	1.006,46	427,16	2	2.837,3
Famílias beneficiadas no programa Bolsa Família **	2.919	4.334,93	9.334,91	475	200.124
*Dummy* (1, se município remoto; 0, caso contrário, segundo a média estadual de tipologia por localização)	2.919	0,18	0,38	0	1

ECG: eletrocardiograma; DP: desvio padrão; SISVAN: Sistema de Vigilância Alimentar e Nutricional; SUS: Sistema Único de Saúde.

Fonte: elaborado pelos autores a partir da análise dos dados no software RStudio, versão 4.1.2 (https://rstudio.com/).

* Por município;

** Por município, por local de residência.

A Tabela 2 mostra as internações motivadas por um conjunto de doenças cardiovasculares, ocorridas de 2014 a 2020 na Bahia. A hipertensão arterial apresenta a maior ocorrência média por município (51,45), seguida por acidente vascular e outras doenças isquêmicas do coração (43,9). A hipertensão arterial é um importante fator de risco para as doenças cardiovasculares e se relaciona diretamente com o AVC. Os nove agravos cardiovasculares estudados ocorrem em 100% do território baiano, com ao menos um acometimento/ano/município (mesmo para as doenças coronárias reumatológicas, que apresentam a menor média de ocorrência).

**Tabela 2:** Descritivo das variáveis de controle de agravos para a população dos municípios do Estado da Bahia, Brasil (2014 a 2020).

Variáveis *	Observações	Média	DP	Mínimo	Máximo
Internações por hipertensão arterial sistêmica	2.893	51,45	187,84	1	3.983
Internações por doença arterial periférica	1.575	7,47	24,31	1	314
Internações por doença coronariana	1.882	5,64	26,53	1	537
Internações por doenças cardíacas reumáticas	1.332	3,15	12,55	1	211
Internações por diabetes mellitus	2.867	29,65	70,35	1	1.359
Internações por arritmia cardíaca	2.377	8,45	41,60	1	877
Internações por acidente vascular cerebral e outras doenças isquêmicas do coração	2.907	43,90	177,40	1	3.846
Internações por infarto agudo do miocárdio	2.687	17,04	69,22	1	1.670
Internações por insuficiência cardíaca	2.879	36,75	97,94	1	2.083

DP: desvio padrão.

Fonte: elaborado pelos autores a partir da análise dos dados no software RStudio, versão 4.1.2 (https://rstudio.com/).

* Todos os dados provenientes do Sistema de Informações Hospitalares (SIH), e por município e local de residência.

A Tabela 3 mostra que, para o período de análise, cada município teve os leitos de internação ocupados por um total de 1.208 dias, em média, a um custo médio anual de R$ 312.898,40. O custo médio individual foi de R$ 1.612,86, com uma média de internação de até aproximadamente seis dias de permanência. A distribuição por gênero foi equivalente (49,1% para masculino e 50,9% para feminino) e as variáveis que indicam raça/cor apresentam, em geral, um déficit de aproximadamente 27% nas observações, o que sugere ser um indicador negligenciado nos registros de atendimento em saúde. Apesar da proximidade no número médio de internações entre pessoas autodeclaradas de cor branca e de cor preta; para estas o número máximo de internações e o desvio padrão chegam a ser quase três vezes maiores por município. Há predominância das internações por doenças cardiovasculares entre a faixa etária acima de 59 anos (média de 98 internações), em relação à faixa etária entre 30 a 59 anos (média de 63 internações) por município. A taxa de mortalidade média está em torno de 10%, podendo chegar a quase 50% para alguns municípios, com 16 óbitos em média, podendo ultrapassar mil óbitos por ano em alguns casos.

**Tabela 3:** Descritivo das variáveis de controle socioeconômicas para os municípios do Estado da Bahia, Brasil (2014 a 2020).

Variáveis *	Observações	Média	DP	Mínimo	Máximo
Dias de permanência **	2.919	1.208,66	8.794,71	3	208.138
Serviços de atenção cardiovascular com atendimento pelo SUS ***	2.919	166,97	687,2	1	15.207
Valor total das internações (R$) **	2.919	312.898,4	2.082.873	707,46	4.773.131
Valor médio de internações (R$) **	2.919	1.612,86	882,79	261,67	10.318,73
Número médio de dias de permanência **	2.919	5,69	2,14	1,6	41,8
Internações do sexo masculino **	2.919	82,00	314,00	1	6.993
Internações do sexo feminino **	2.917	85,02	374,23	1	8.337
Internações de pessoas na faixa etária entre 30 e 59 anos **	2.914	63,51	269,52	1	6.341
Internações de pessoas na faixa etária acima de 59 anos **	2.917	98,35	391,38	1	8.973
Internações de pessoas autodeclaradas brancas **	2.208	12,18	37,30	1	717
Internações de pessoas autodeclaradas pretas **	2.122	12,24	88,48	1	2.098
Taxa de mortalidade, por 100 mil habitantes **	2.819	10,04	5,78	0,39	47,37
Óbitos **	2.819	16,13	58,97	1	1.183

DP: desvio padrão; SUS: Sistema Único de Saúde.

Fonte: elaborado pelos autores a partir da análise dos dados no software RStudio, versão 4.1.2 (https://rstudio.com/).

* Todos os dados provenientes do Sistema de Informações Hospitalares (SIH);

** Por município, por local de residência;

*** Por município.

Os coeficientes estimados no modelo *diff-in-diff* estão na Tabela 4, todos com alto grau de significância. As variáveis de controle sugerem que os municípios com adesão ao serviço de telecardiologia conseguiram uma redução de seis internações por doenças cardiovasculares a cada ano adicional. Além disso, há reduções de 3,26 internações por doenças cardiovasculares entre as pessoas com famílias beneficiadas no programa Bolsa Família, e de 3,08 entre aqueles que possuem o serviço especializado. Os municípios que disponibilizam a realização convencional do exame de ECG contribuem para a redução de 7,31 internações por doenças cardiovasculares. Entretanto, há um aumento de 7,66 internações entre a faixa etária de 30 a 59 anos e aumento de 5,34 internações entre homens, a cada ano adicional.

**Tabela 4:** Estimativas do modelo *diff-in-diff* para os municípios do Estado da Bahia, Brasil (2014 a 2020).

Variáveis	Coeficiente	P<|t| *
Internações por doenças cardiovasculares **	-6,261	0,004
Internações por arritmia cardíaca **	1,236	0,000
Internações de pessoas na faixa etária entre 30 e 59 anos **	7,660	0,000
Internações por acidente vascular cerebral e outras doenças isquêmicas do coração **	6,636	0,000
Famílias beneficiadas no programa Bolsa Família **	-3,269	0,009
Serviços de atenção cardiovascular com atendimento pelo SUS ***	-3,083	0,000
Internações por doença coronariana **	1,263	0,000
Internações por doenças cardíacas reumáticas **	-1,151	0,049
Internações por diabetes mellitus **	-1,399	0,000
Exames de ECG convencionais realizados **	-7,319	0,008
Internações por hipertensão arterial sistêmica **	4,262	0,000
Internações por infarto agudo do miocárdio **	3,496	0,000
Internações por insuficiência cardíaca **	5,178	0,000
Internações do sexo masculino **	5,341	0,000
Número médio de dias de permanência **	-3,395	0,007
Valor médio de internações (R$) **	-2,744	0,000
Internações de pessoas autodeclaradas pretas **	-4,665	0,007
Taxa de mortalidade por 100 mil habitantes **	-9,545	0,000

ECG: eletrocardiograma; SUS: Sistema Único de Saúde.

Fonte: elaboração própria.

* Significante ao nível de 5%, R^2^ ajustado: 0,9986;

** Por município, por local de residência;

*** Por município.

Na análise dos agravos, o modelo sugeriu uma diminuição de 1,15 internações por doenças cardíacas reumatológicas e de 1,39 entre as pessoas que vivem com diabetes. Entretanto, há um aumento de 1,23 internações por arritmia cardíaca, de 6,63 por acidente vascular cerebral e outras doenças isquêmicas do coração, 1,26 nas internações por doença coronariana e de 4,26 entre as pessoas com hipertensão arterial sistêmica, nos municípios com adesão ao serviço. O mesmo padrão de aumento nas internações foi encontrado para o infarto agudo do miocárdio (3,49) e para a insuficiência cardíaca (5,17), a cada ano adicional. Contudo, observa-se a diminuição de 4,66 nas internações por doenças cardiovasculares entre as pessoas autodeclaradas pretas, redução de 3,39 na média de dias de internamento e de R$ 2,74 no valor médio por internação, além de uma redução de 9,54% na taxa de mortalidade, a cada ano adicional em cada município com adesão ao serviço.

## Discussão

Apesar de a prevenção primária ser viável, utilizando-se da investigação, da detecção e do manuseio dos fatores de risco, a crescente incidência das doenças cardiovasculares tem representado graves implicações na qualidade de vida das pessoas e na morbidade hospitalar, além de representarem grande impacto na economia dos sistemas de saúde e na seguridade social ^24^.

Parte desse impacto também pode ser verificada por meio dos resultados deste estudo, no que se refere ao indicativo de aumento nas internações por doenças cardiovasculares na população economicamente ativa, na faixa etária de 30 a 59 anos, principalmente entre os homens, cuja busca por serviços de saúde é menos frequente, tornando a dimensão de gênero um determinante do processo saúde-doença na população masculina ^25^. A PNS de 2019 mostra que a proporção de homens que consultaram um médico foi inferior à de mulheres (69,4% e 82,3%, respectivamente) ^9^.

Esse fato mostra que os sistemas de telecardiologia implantados em municípios remotos apresentam grande potencial para a ampliação do acesso à assistência de saúde especializada, favorecendo a prevenção e o diagnóstico precoce, contribuindo, dessa maneira, para a redução das internações gerais por doenças cardiovasculares. Esse comportamento também pode ser percebido entre as pessoas com acompanhamento das condicionalidades de saúde do programa Bolsa Família na atenção primária à saúde. Nos municípios em que são oferecidos serviços especializados em cardiologia, observa-se redução nas internações.

Apesar de não formalmente avaliado, um estudo que analisou a implantação do serviço de telecardiologia no Estado de Minas Gerais corrobora com a facilitação do acesso ao ECG e a avaliação cardiológica especializada, considerando que a maioria dos serviços de cardiologia e cirurgia cardiovascular está concentrada nos grandes centros urbanos, contexto que limita o acesso da população de cidades menores e remotas a métodos diagnósticos básicos ^26^. Sendo assim, assegurar a disponibilidade e a acessibilidade à saúde, conforme demandas da população, é um dos grandes desafios para o sistema de saúde implementado no Brasil, em um cenário no qual há escassez de médicos, sobretudo de especialistas, e má distribuição geográfica entre os níveis de atenção ^27^.

É nesse sentido que o aumento inicial nas internações de alguns agravos sugeridos pelo estudo, principalmente as doenças isquêmicas do coração, a insuficiência cardíaca e o infarto agudo do miocárdio, podem estar relacionados, provocando um represamento de casos não diagnosticados. A disponibilidade de um serviço em uma região em que ocorrem limitações na acessibilidade à assistência para determinados grupos populacionais pode sinalizar para o SUS que é necessário traçar estratégias para melhorar a qualidade do cuidado prestado ^28^.

Mendes ^29^ chama a atenção para a crescente participação das condições crônicas na situação de saúde e do papel que o acesso aos serviços tem na identificação das necessidades do cuidado. Mendes ^29^ (p. 11) ainda define o acesso à saúde como: “...*a oportunidade de buscar e obter serviços de saúde apropriados em situações de necessidades percebidas de cuidado. Assim, o acesso resulta de uma interface entre as características das pessoas, das famílias, dos ambientes físicos e sociais e as características do sistema de atenção à saúde, das organizações que o compõem e dos prestadores de serviços*”.

Outro resultado que se sobressai da estimação refere-se à dimensão raça/cor. Este estudo mostra uma redução nas internações das pessoas autodeclaradas de cor preta, ressaltando que a telecardiologia pode ser um importante instrumento no combate às iniquidades em saúde, oportunizando o acesso ao cuidado especializado a um grupo populacional que seria facilmente negligenciado pelos modelos assistenciais praticados. A própria base de dados utilizada demonstra que essa informação é pouco requisitada durante o preenchimento das fichas de atendimento nos sistemas de informação em saúde vigentes. Como revelou a PNS de 2019, o diagnóstico médico para doenças cardiovasculares foi menor entre pessoas autodeclaradas pretas, bem como a solicitação do exame de ECG durante as consultas com médico especialista.

O percentual de pessoas que nunca aferiram a pressão arterial foi duas vezes maior em pessoas autodeclaradas pretas do que em autodeclaradas brancas, além de sofrerem mais por limitações intensas provocadas por alguma doença cardíaca, apesar de representarem aproximadamente 70% do público total atendido pelo SUS ^9^, o que pode sinalizar que o direito à saúde da população negra é violado, fato conhecido como racismo institucional. Ademais, o acesso precário aos serviços de saúde pública favorece o acometimento de diversos outros agravos, físicos ou mentais ^25^. Portanto, a não inclusão das questões étnico-raciais e de racismo na coleta das informações de saúde para a análise e a divulgação, bem como o não investimento em ações e programas específicos, podem ser elencadas como práticas discriminatórias ^30^.

Quanto à prevenção de agravos e diagnóstico de doenças cardiovasculares, o estudo sugere eficácia na identificação precoce dos sinais de comprometimento do quadro clínico, possibilitando intervenção a tempo, reduzindo o período de tratamento em ambiente hospitalar, bem como uma redução na média dos dias de internamento.

Portanto, a telecardiologia tem demonstrado sua utilidade no controle e na vigilância de fatores de risco, emergindo como uma opção viável que possibilita uma intervenção mais efetiva baseada na prevenção. Além disso, ela contribui para o diagnóstico, o tratamento e o acompanhamento em cardiologia, com o propósito de fornecer cuidados de maior qualidade à população e promover uma atenção à saúde de maneira integral ^31^.

Algumas iniciativas têm sido implementadas no Serviço de Atendimento Móvel de Urgência (SAMU), nas quais a transmissão pré-hospitalar do ECG para o cardiologista de plantão reduz o tempo gasto até a realização do cateterismo/angioplastia coronariana, além de permitir o uso prévio de fármacos trombolíticos, possibilitando o tratamento imediato e reduzindo as chances de sequelas, como a insuficiência cardíaca ^32^.

Pacientes assistidos em Unidades de Pronto Atendimento (UPA) no Rio de Janeiro foram objeto de uma análise que avaliou o emprego da telecardiologia para auxiliar no diagnóstico diferencial de dor torácica. Essa análise categorizou a aplicação dessa tecnologia como viável para o diagnóstico diferencial. Adicionalmente, foi capaz de identificar os casos que realmente necessitavam de transferência para unidades terciárias de referência, prevenindo a ocupação imprópria de leitos hospitalares em 13% dos pacientes assistidos, o que resultou em uma redução significativa nos gastos públicos ^33^.

Essa mesma redução nos custos também foi verificada neste estudo, no qual o resultado de uma redução no valor médio por internação corrobora os resultados do estudo de custo-benefício de Andrade et al. ^34^, em Minas Gerais, em que foi demonstrado o benefício econômico na realização do ECG entre os municípios de pequeno porte, com sistema de telecardiologia implantados na atenção primária à saúde.

Em resumo, a telecardiologia, aplicada às ações de promoção da saúde e prevenção primária e secundária de doenças cardiovasculares, apresenta potencial para contribuir com uma significativa redução de custos, atrelada à intervenção precoce, possibilitando a redução de consultas ao especialista, internações por complicações clínicas severas e entradas nas unidades de urgência, dessa maneira, reduzindo os custos e ampliando a eficiência dos sistemas de saúde ^32^.

A telecardiologia oferece muitas possibilidades de melhorias na prevenção e no controle de fatores de risco, redução do tempo de diagnóstico com o fornecimento do laudo correto do eletrocardiograma e apoio à decisão terapêutica, além do tratamento oportuno para as doenças cardiovasculares, com potencial impacto sobre a morbimortalidade relacionada ^16^.

## Considerações finais

Os resultados sugerem uma significativa redução nas internações gerais motivadas por doenças cardiovasculares nos municípios onde houve a implantação do serviço de telediagnóstico em cardiologia.

Ainda que os dados revelem uma alta sensibilidade para algumas das internações analisadas, particularmente as associadas ao AVC e outras patologias isquêmicas do coração, esse fenômeno pode ser um indicativo de um desequilíbrio entre a demanda reprimida e a oferta insuficiente de serviços nos municípios especialmente evidente entre os municípios com dificuldades no acesso a cuidados especializados em cardiologia.

As diminuições observadas na taxa de mortalidade, no tempo médio de internações por doenças cardiovasculares e no potencial para a redução de custos, evidenciam a relevância do acesso a exames para o diagnóstico diferencial e a intervenção precoce. Esses são elementos cruciais para a melhoria da qualidade de vida dos indivíduos e para a expansão do acesso de maneira equânime.

As questões de raça tornaram-se aparentes, mesmo não sendo alvo deste estudo: existe uma subnotificação para todos os agravos estudados, resultando em prognósticos mais severos para as condições do miocárdio quando diagnosticadas. Portanto, a telecardiologia surge como um instrumento potencialmente eficaz para mitigar as desigualdades, uma vez que demonstrou ser significativa na expansão do acesso a serviços médicos especializados e na redução das internações entre indivíduos que se autodeclaram pretos e entre os participantes de programas sociais.

Diante dos achados apresentados, conclui-se que a telecardiologia, no âmbito do exame eletrocardiográfico, exerce uma função primordial na saúde coletiva, evidenciando um expressivo impacto na preservação da vida e na diminuição do coeficiente de mortalidade. Nesse sentido, torna-se imprescindível incentivar a adoção dessa tecnologia em todo o território nacional, como um recurso indispensável da Estratégia de Saúde Digital e para a elaboração de uma Política Nacional de Saúde Digital no SUS.

A literatura sugere a necessidade de um incremento nos investimentos para a implementação dessa tecnologia na atenção primária à saúde, na formação de profissionais para os serviços de telecardiologia, assim como na condução de estudos mais robustos que sistematizem seus impactos em diversos grupos populacionais. Grande parte da literatura internacional consultada fundamenta suas descobertas no uso da telemedicina para o monitoramento de eventos após ocorrências cardíacas ou em processos de reabilitação, em detrimento a ações de prevenção.
